# Associations of volatile organic compounds with accelerated epigenetic aging in the lungs of smokers and electronic cigarette users

**DOI:** 10.1016/j.scitotenv.2025.179792

**Published:** 2025-05-30

**Authors:** Ajmal Khan, Carson Richardson, Joseph P. McElroy, Amarnath Singh, Daniel Y. Weng, Sahar Kamel, Sarah A. Reisinger, Mark D. Wewers, Peter G. Shields, Min-Ae Song

**Affiliations:** a Division of Environmental Health Sciences, College of Public Health, The Ohio State University, Columbus, OH, USA; b Center for Biostatistics, College of Medicine, The Ohio State University Wexner Medical Center, Columbus, OH, USA; c Comprehensive Cancer Center, The Ohio State University and James Cancer Hospital, Columbus, OH, USA; d Pulmonary and Critical Care Medicine, Department of Internal Medicine, The Ohio State University, Columbus, OH, USA; e Center for Tobacco Research, The Ohio State University Comprehensive Cancer Center, Columbus, OH, USA

**Keywords:** Epigenetic aging, Smoking, Electronic cigarettes, Volatile organic compounds, Lungs

## Abstract

**Background:**

Cigarette smoke and electronic cigarettes (EC) contain volatile organic compounds (VOCs) that can irritate the respiratory tract and affect pulmonary health. No studies have reported relationships between VOCs from smokers and EC users and pulmonary cellular behavior, such as lung biological aging.

**Methods:**

Using urinary total nicotine equivalent (TNE) and propylene glycol, the primarily *E*-liquid constituent, the tobacco use status of smokers (SM, *n* = 13), EC users (*n* = 12), and never-smokers (NS, *n* = 31) was confirmed. In the lungs of SM and EC, we assessed methylation age (mAge) estimates and cellular senescence-related gene expression. Ten urinary metabolites of VOCs with and without adjusting for nicotine intake, TNE, were associated with mAge estimates and gene expression.

**Results:**

Compared to EC users, SM had a significantly higher level of TNE-adjusted metabolites of acrylonitrile and 1,3-butadiene, while showing a lower level of a metabolite of benzene. TNE-adjusted metabolites of 1,3 butadiene, ethylene oxide, and acrylonitrile were overall positively associated with Pheno-mAge (an epigenetic clock for lifespan/mortality), with different patterns of the associations by smoking group. *IFNG* and *STAT1* showed an overall positive association with TNE-adjusted metabolites of propylene oxide and acrylamide, with a stronger association in SM compared to EC. Without controlling for TNE, a benzene metabolite was the only significant associated with *POU5F1*.

**Conclusions:**

This is the first study reporting associations between smoking/EC-related VOCs and lung biological aging with strong associations in SM, supporting the need for further research on the roles of VOCs-related epigenetic aging and cell senescence genes in pulmonary diseases.

## Graphical Abstract

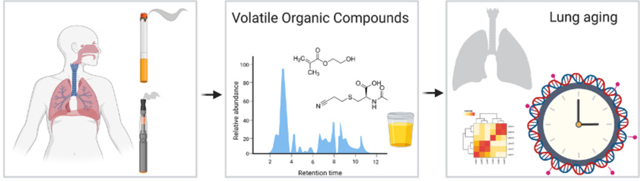

## 1. Introduction

The tobacco epidemic is considered one of the biggest global health threats (St Claire et al., 2020). Despite a reduction in current smoking rates, 1.3 billion individuals worldwide continue to smoke (WHO, 2023). In the United States, cigarette smoking is the leading cause of preventable disease and death (Services, 2020), with over 16 million Americans living with a smoking-related disease.

Cigarette smoke is a complex mixture that contains volatile organic compounds (VOCs), some of which can cause airway irritation (Rumchev et al., 2007) and is known to be linked to pulmonary diseases (Hussain et al., 2024; Janssens et al., 2020; Lv et al., 2023). When cigarettes are burned, significant amounts of toxic VOCs are generated due to the incomplete combustion of tobacco (Pazo et al., 2016). These harmful substances are inhaled into the lungs, released into the bloodstream, metabolized primarily in the liver, and yield several toxic metabolites excreted in the urine (Li et al., 2021). Of them, 1,3-butadiene, benzene, acrylamide, styrene, and benzyl chloride are known to be carcinogenic or potentially carcinogenic (IARC, 2015, 2019). Altered biological pathways implicated in VOC-induced pathogenesis include impaired cellular signaling (Dezest et al., 2017), cellular apoptosis (Sayyed et al., 2022), oxidative stress (Kim et al., 2011), inflammation (Kwon et al., 2018), and mutations in DNA repair genes (Dezest et al., 2017).

Smoking and smoking-related lung diseases are associated with significant epigenetic changes, particularly in DNA methylation at CpG dinucleotides (CpGs) (Eriksson Strom et al., 2022; Seiler et al., 2020) which can be influenced by smoking (Li et al., 2024). Specifically, an animal experimental study showed DNA methylation changes followed by exposure to cigarette smoke, leading to lung inflammation (Seiler et al., 2020). Clinical studies have also identified smoking-associated CpGs across different tissue types with lung tissue showing smoking-related CpGs enriched in xenobiotic and cancer-related genes (Li et al., 2024). In addition, in patients with smoking-related diseases (i.e., chronic obstructive pulmonary disease [COPD]), distinct methylation patterns were found in the lungs (Eriksson Strom et al., 2022).

In recent years, epigenetic age acceleration (also known as accelerated epigenetic aging), reflecting faster biological aging than expected based on chronological age, has emerged as a biological tool for understanding chronic diseases and risks, including smoking-related pulmonary diseases. Epigenetic age acceleration is measured by DNA methylation (mAge), which has been linked to smoking and smoking-related diseases, and aging is a major risk factor for chronic pulmonary diseases (Brandsma et al., 2017; Chen and Wu, 2020). Longitudinal cohort studies showed a prospective association of blood mAge acceleration and the incidence of COPD (Breen et al., 2020), suggesting its potential use for risk prediction. Further, mAge acceleration was observed in the lungs of smokers (Song et al., 2023; Wu et al., 2019). However, the potential impacts of specific cigarette toxicants (i.e., VOCs) on biological aging remain unknown. Studying these relationships in target organs of diseases (i.e., lungs) is particularly important to understand potential contributions of mAge acceleration in disease pathogenesis.

Several VOCs are also found in electronic cigarettes (EC) (Papaefstathiou et al., 2020), although those levels are lower than those found in cigarette smoking (St Helen et al., 2020). EC use has raised public concern, given its popularity as an alternative to smoking to reduce or quit tobacco consumption and appealing to youth and young adults (Polosa et al., 2022), with limited objective evidence of its safety for long-term use (Pierce et al., 2020). While epigenetic alterations caused by cigarette smoking are well-documented (Seiler et al., 2020; Song et al., 2023; Yang et al., 2019), there are mixed findings regarding the epigenetic modifications by EC use (Anic et al., 2022; Herzog et al., 2024; Richmond et al., 2021; Song et al., 2023). Interestingly, our bronchoscopy study of EC users and smokers indicated faster epigenetic aging than expected in both groups compared to never-smokers (Song et al., 2023), but we do not know the potential effects of specific smoking and EC-related VOCs on lung biological aging.

Biomarker studies of exposure and effects can provide insights into internal exposure to toxicants (i.e., VOCs) related to cigarette smoke and EC and their potential harmful impacts on diseases, which are potentially via biological aging. VOCs are implicated in pulmonary disease pathogenesis, and aging is one of the most well-known risk factors for lung diseases. Thus, we investigated the overall relationships between smoking/EC-related VOCs and lung biological aging, measured by epigenetic clocks and cellular senescence-related gene expression, and further potential different patterns of the relationships by smoking group, with and without adjusting for nicotine intake.

## 2. Materials and methods

### 2.1. Study participants

Recruitment and study design were previously documented in detail (Mori et al., 2022; Song et al., 2020). Briefly, in 2015–2019, local print and television media were used to recruit study participants to understand the pulmonary biological impact of smoking and EC use; all were healthy adults aged 21–30 years willing to have a bronchoscopy. The participants who were regular users of marijuana and/or those who had underlying medical conditions such as COPD, cancer, autoimmune disorders, etc., were excluded. EC users (*n* = 12) puffed nicotine-containing EC daily for more than one year and had not smoked a cigarette for more than five months. Never-smokers (NS, *n* = 31) smoked *<*100 cigarettes in their lifetime and not smoked or puffed for at least one year before participation. Exclusive smokers (SM, *n* = 13) smoked >10 cigarettes per day for more than six months and had not regularly used EC for at least one year. Smoking status was verified using a Nicalert cotinine test strip (Nymox Pharmaceutical Corporation, St. Laurent, QC, Canada) at orientation and bronchoscopy visits.

### 2.2. Ethical approval, participants' consent, and bronchoscopy

The Institution Review Board (IRB) of The Ohio State University (OSU) approved the study (Protocol: 2015C0088, ClinicalTrials.gov: NCT02596685). Written informed consent was obtained from each participant. Bronchial epithelial brushing samples from either the right or left mainstem bronchus were collected during a research bronchoscopy at The Ohio State University Clinical Research.

### 2.3. Epigenetic age calculation

Epigenetic age estimates were generated from lung bronchial epithelial cells using genome-wide DNA methylation as determined by Infinium Methylation EPIC BeadChip (Illumina, San Diego, CA) as reported in our previous studies (Song et al., 2020; Song et al., 2023). Partek Genomic Suite ™ 6.6 (St. Louis, MO) was employed to normalize the raw data files by subset quantile Within Array Normalization (Song et al., 2023). Probes with a detection *P >* 0.05 were excluded from the analysis. As described in our previous study (Song et al., 2023), mAge estimates for DNA methylation-based chronological aging (Horvath-mAge), lifespan and mortality risks (GrimAge and Pheno-mAge), and DNA methylation-based telomere length (TL-mAge) were calculated using Horvath's New Methylation Age Calculator (https://dnamage.genetics.ucla.edu/ new) with advanced analysis (Lei et al., 2020; Lu et al., 2019). Using the calculator's internal normalization method, SWAN normalized β-values were processed. The acceleration of mAge (mAA) estimates were obtained as the residuals calculated by a linear mAge model on chronological age (Horvath, 2015).

### 2.4. Cellular senescence-related genes

Cellular senescence-related gene expressions were captured from gene expression profiling using the GeneChip ® Human Transcriptome Array 2.0 (Affymetrix Inc., Santa Clara, CA). CEL files were log_2_ transformed and underwent quantile normalization in Partek (Song et al., 2020). We studied the 1133 genes listed from ‘CellAge: The Database of Cell Senescence Genes’ (https://genomics.senescence.info/cells/) (Avelar et al., 2020; Chatsirisupachai et al., 2019).

### 2.5. Urinary metabolites of VOCs

Liquid chromatography-tandem mass spectrometry LC-MS/MS was employed to detect the level of ten mercapturic acid metabolites of VOCs in the urine samples (St Helen et al., 2020). Briefly, LC-MS/MS analyses were performed with an Agilent (Palo Alto, CA) 1200 HPLC coupled with a Thermo-Finnigan (San Jose, CA) TSQ Quantum Ultra triple-stage quadrupole mass spectrometer, with an atmospheric pressure chemical ionization (APCI) source. The chromatographic separation was performed using gradient elution on an analytical column (Phenomenex Synergi Polar-RP column, 150 mm × 4.6 mm, 4 μm, Phenomenex Inc. Torrance, CA). The ten mercapturic acid metabolites measured were phenyl mercapturic acid (PMA) (a metabolite of benzene), 4-hydroxy-2-buten-1-yl-mercapturic acid (MHBMA3) (a metabolite of 1,3-butadiene), 2-hydroxy-3-buten-1-yl-mercapturic acid (MHBMA1,2) (a metabolite of 1,3-butadiene), 2-hydroxyethylmercapturic acid (HEMA) (a metabolite of ethylene oxide), methyl mercapturic acid (MMA) (a metabolite of methylating agent), cyanoethyl mercapturic acid (CNEMA) (a metabolite of acrylonitrile), 3-hydroxypropyl mercapturic acid (3-HPMA) (a metabolite of acrolein), 2-hydroxypropyl mercapturic acid (2-HPMA) (a metabolite of propylene oxide), *N*-acetyl-S-(2-carbamoylethyl)-L-cysteine (AAMA) (a metabolite of acrylamide), and 3-hydroxy-1-methylpropylmercapturic acid (HPMMA) (a metabolite of crotonaldehyde) (Supplementary Table 1). Urinary biomarkers are reported as a normalized ratio to urinary creatinine (Cr). To assess the nicotine intake-adjusted biomarkers among smokers and EC users to control for differences in nicotine exposure, Cr-adjusted metabolites were further normalized using the molar sum of two major nicotine metabolites (Trans-3-hydroxy cotinine and cotinine) as the total nicotine equivalent (TNE).

### 2.6. Statistical analysis

All data analyses were performed using R V 4.4.0 software. One participant from the EC group was excluded from analyses due to implausibly high TNE-adjusted biomarker estimates. This resulted in a final sample of 56 participants, including 13 SM, 12 EC users, and 31 NS. Between-group differences were restricted to SM and EC for TNE-adjusted biomarkers, whereas all three groups were included in the creatinine-adjusted (Cr-adjusted) biomarkers. Between-group differences in biomarker estimates were tested using log_10_ transformed values in both univariable and multivariable models. Multivariable models were adjusted for race, gender, and chronological age. For Cr-adjusted biomarkers (SM, EC users, and NS), ANOVA followed by pairwise comparisons using Tukey's Honestly Significant Difference (HSD) was utilized. For TNE-adjusted biomarkers (SM and EC users), between-group differences were tested using a two-sample *t*-test or linear regression when covariables were included.

Associations with DNA methylation age (mAge) estimates, their acceleration (mAA), and gene expression with biomarkers were assessed using separate linear regression models. The model for mAge was mAge ~ smoking status + race + gender + chronological age + biomarker + (smoking status x biomarker), while the model for mAA was mAA ~ smoking status + race + gender + biomarker + (smoking status x biomarker). The model for gene expression was Gene ~ smoking status + race + gender + chronological age + biomarker + (smoking status x biomarker). All models used log_10_-transformed biomarker estimates. Each model was included in ANCOVA to test the significance of the interaction effect in models. Within-group analyses were performed for any significant biomarkers (from main or interaction effects). To control for multiple testing, a False Discovery Rate (FDR) was implemented, and an FDR *<* 0.1 was considered significant.

## 3. Results

### 3.1. Characteristics of study participants

The characteristics of the study participants (13 SM, 12 EC users, and 31 NS) and product use history are summarized in Table 1. Overall, the mean chronological age of the participants was 26.4 years. A majority of smokers (84.6 %) and EC users (69.2 %) were male, while males accounted for 41.9 % of NS. Smokers had a median reported smoking history of 9 years and 20 cigarettes smoked per day. EC users, including 10 former smokers, had a median reported vaping history of 3 years. The median puff per day was 100, and the median e-liquid daily use was 7.5 ml. The median TNE for SM (19.5 pg/mg Cr) and EC users (15.9 pg/mg Cr) were considerably higher compared to NS (0.003 pg/mg Cr). EC users had the highest median level of propylene glycol, the primary *E*-liquid constituent, at 28.5 mg/ml, followed by SM at 6.6 mg/ml and NS at 1.98 mg/ml.

### 3.2. Comparisons of urinary VOC metabolites among smokers and EC users

When nicotine intake was adjusted using TNE (Table 2), compared to EC users, SM had a significantly higher level of metabolites of acrylonitrile (CNEMA, 1.8-fold, *P* = 1.00E-03) and 1,3-butadiene (MHBMA 1,2, 1.7-fold, *P* = 8.00E-03), while showing a significantly lower level of a metabolite of benzene (PMA. − 1.3-fold, *P* = 2.50E-02).

### 3.3. Associations between urinary VOC metabolites with methylation age estimates in SM and EC users

To understand the comprehensive relationships between urinary smoking and EC-related VOC metabolites and mAge estimates and their acceleration (mAA), we utilized urinary VOC data with and without considering nicotine intake in separate models.

**Fig. 1.**
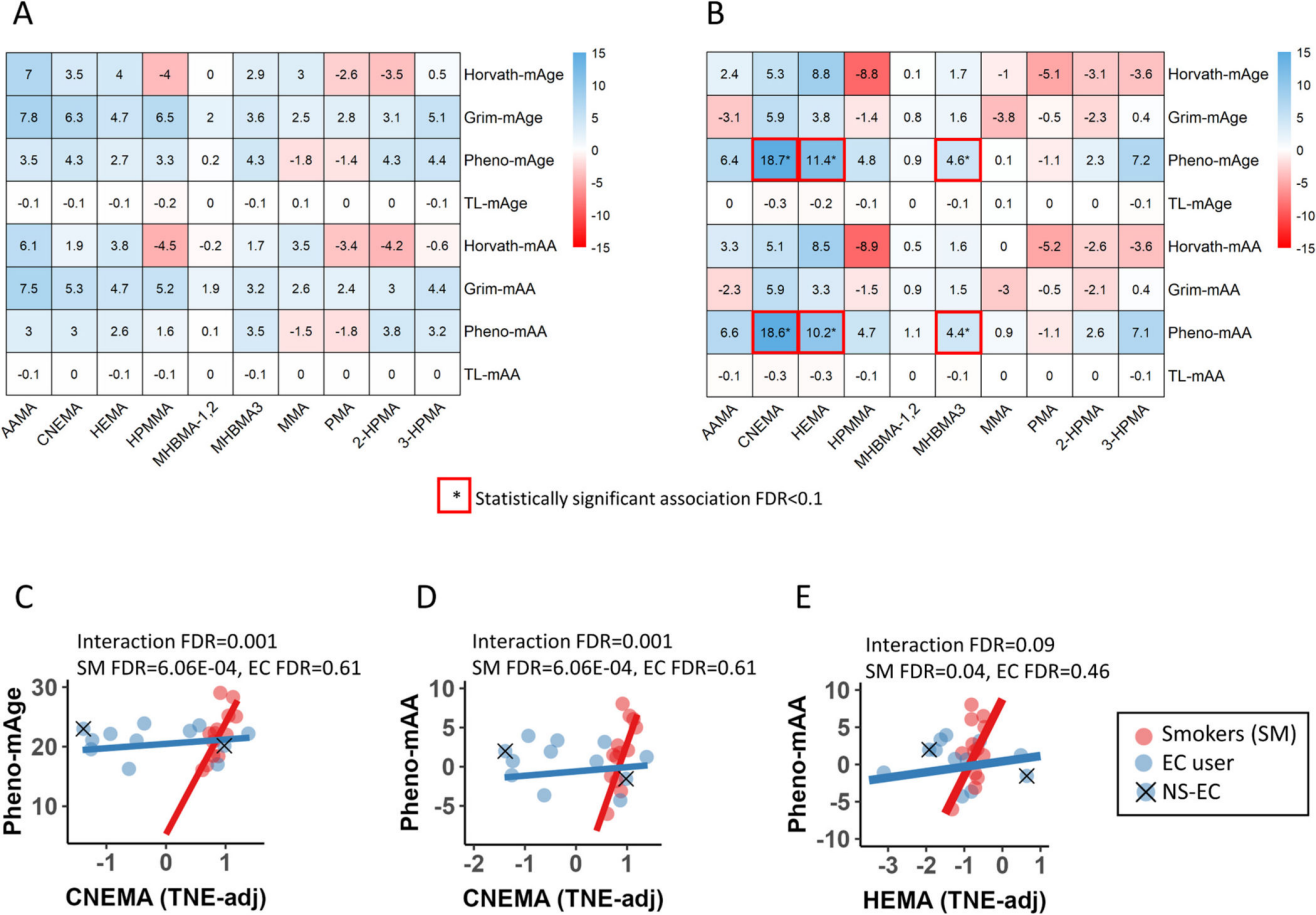
Associations of methylation age (mAge), and accelerated mAge (mAA) estimates with Cr-adjusted and TNE-adjusted VOCs. **A.** Heatmap displaying the associations between mAge, and mAA measures with Cr-adjusted VOCs. The scale bar displays the β-values ranging from − 15 (red) to 15 (blue). **B.** Heatmap showing the associations between mAge, and mAA measures with TNE-adjusted VOCs. Coefficients from linear regression (mAA ~ VOC + smoking_status + race + gender + VOC*smoking_status) & (mAge ~ VOC + smoking_status + chronological_age + race + gender + VOC*smoking_status). The scale bar indicates the β-values ranging from − 15 (red) to 15 (blue). The β-values indicated by the red lines have a statistically significant association with FDR *<* 0.1. **C. C.** Regression plot shows interaction association between TNE-adjusted CNEMA (x-axis) and Pheno-mAge estimate (y-axis). The plot was produced via linear regression (mAge ~ VOC + smoking_status + chronological_age + race + gender + VOC*smoking_status). **D-E.** Regression plots show interaction associations between TNE-adjusted VOCs (x-axis) and Pheno-mAA estimates (y-axis). Both plots were produced via linear regression (mAA ~ VOC + smoking_status + race + gender + VOC*smoking_status). Each dot represents individual, electronic cigarette users (EC, blue), and cigarette smokers (SM, red).

There were no overall significant associations between VOC metabolite (Cr-adjusted, without nicotine-intake adjustment) and mAge and mAA estimates, independent of smoking groups, race, and gender (Fig. 1A, Supplementary Table 2). However, with TNE-adjusted metabolites (Fig. 1B, Supplementary Table 3), metabolites of acrylonitrile (CNEMA, FDR = 7.05E-04 and FDR = 7.05E-04, respectively), ethylene oxide (HEMA, FDR = 0.07 and FDR = 0.06, respectively), and 1,3 butadiene (MHBMA3, FDR = 0.07 and FDR = 0.09, respectively) had overall significantly positive associations with Pheno-mAge and Pheno-mAA. In addition, a significant interaction was identified between a TNE-adjusted metabolite of acrylonitrile (CNEMA) and smoking group (smoking x biomarker) for both Pheno-mAge and Pheno-mAA (FDR = 0.001 and FDR = 0.001, respectively, Fig. 1C and D, Supplementary Table 3). However, no such significant interactions were found for MHBMA3. Additionally, the association of TNE-adjusted metabolite of ethylene oxide (HEMA) and Pheno-mAA was found to differ between SM and EC users (Interaction FDR = 0.09) (Fig. 1E, Supplementary Table 3). Within-group analyses showed these significant interaction effects are driven by the smoking group. There were no statistically significant associations of VOCs with Horvath-mAge, GrimAge, and TL-mAge.

### 3.4. Associations between urinary VOC metabolites and cellular senescence-related gene expression in SM and EC users

We further assessed the potential impact of smoking and EC-related VOCs on cellular senescence-related gene expressions. In the overall analysis using Cr-adjusted metabolites of VOCs (Supplementary Table 4), independent of smoking groups, race, and gender, we identified *POU5F1* level associated with a metabolite of benzene (PMA, FDR = 0.05), and within-group analyses showed this association was driven by smokers (FDR = 0.001 in SM, FDR = 0.49 in EC).

**Fig. 2.**
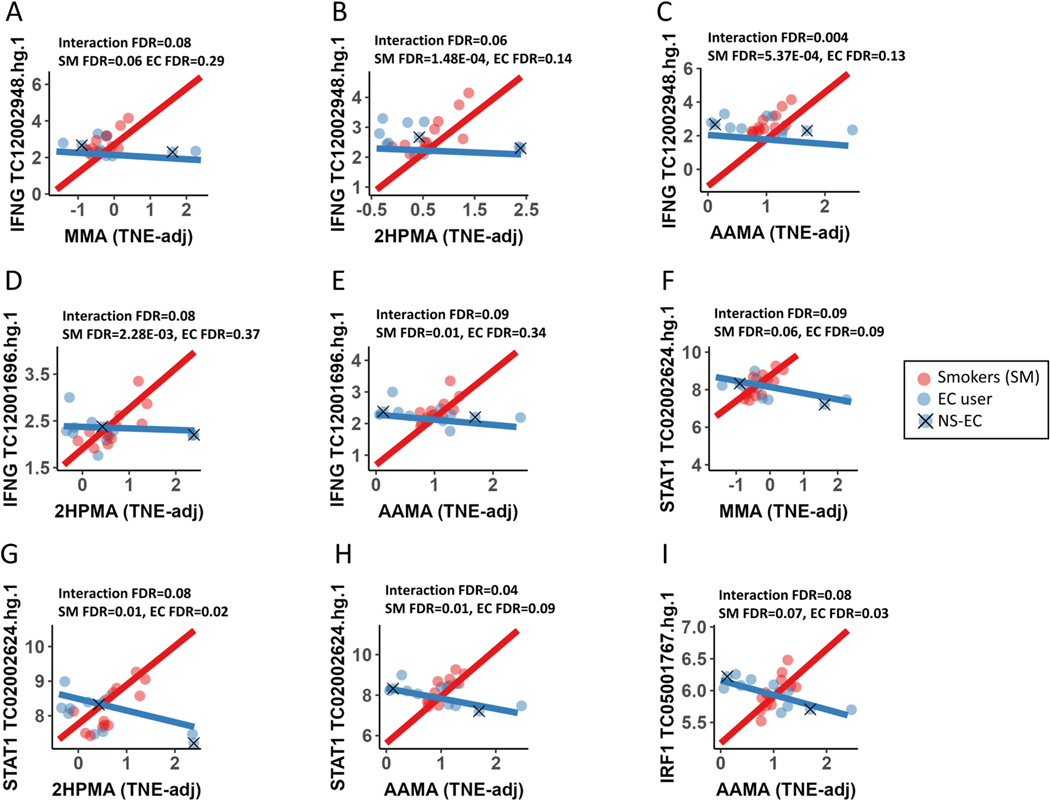
Associations of gene expression with TNE-adjusted VOCs. **A-I.** Regression plots show interaction associations between TNE-adjusted VOCs (x-axis) and gene expression (y-axis). **A-C,** interaction association between IFNG, TC12002948.hg.1 and MMA-TNE, 2HPMA-TNE and AAMA-TNE, **D&E,** interaction association between IFNG, TC12001696.hg.1 and 2HPMA-TNE and AAMA-TNE, **F**–**H,** interaction effect between STAT1, TC02002624.hg.1 and MMA-TNE, 2HPMA-TNE and AAMA-TNE, and **I,** shows interaction effect between IRF1, TC05001767.hg.1 and AAMA-TNE. All models are linear regressions (gene expression ~ VOC + smoking_status + chronological_age + race + gender + VOC*smoking_status). Each dot represents individual, electronic cigarette users (EC, blue), never-smoking EC users (NS-EC, crossed blue), and cigarette smokers (SM, red). Each dot represents individual, electronic cigarette users (EC, blue), and cigarette smokers (SM, red).

With TNE-adjusted metabolites of VOCs (Supplementary Table 5), independent of smoking, we observed metabolites of methylating agent (MMA, FDR = 0.09) and propylene oxide (2-HPMA, FDR = 0.06 or FDR = 0.08) had a significantly positive association with *IFNG* expression. Also, a metabolite of acrylamide (AAMA) showed significant positive associations with *IFNG* (FDR = 0.01) and *STAT1* (FDR = 0.08) expression. All these associations had significant smoking x biomarker interactions with strong associations (Fig. 2A-I), with strong associations driven by smokers (Supplementary Table 5).

## 4. Discussion

This study evaluated the relationships of smoking and EC-related VOCs with pulmonary epigenetic aging and cell senescence-related gene expression. This is the first study to assess several well-known lung epigenetic clocks in the lung, measured by mAge estimates, for biological aging and lung cellular senescence-related genes in relation to a wide range of urinary metabolites of VOCs in young and healthy individuals of SM and EC users. In the current study, we comprehensively evaluated the associations between VOCs and biological aging markers with and without consideration of nicotine intake using TNE-adjusted and Cr-adjusted data, respectively.

Consistent with previous biomarkers of exposure to smoking and EC studies (De Jesus et al., 2020; Gallart-Mateu et al., 2023; Goniewicz et al., 2018), we confirmed the highest Cr-adjusted means of metabolites of VOCs in SM, followed by EC users and NS. We observed that between SM and NS, three metabolites, including CENMA (for acrylonitrile), MHBMA1,2 (for 1,3-butadiene), and PMA (for benzene) showed >10-fold differences. Metabolites of acrylonitrile and 1,3-butadiene also showed significantly higher levels in EC users compared to NS, while the magnitude differences were smaller than that of SM compared to NS (data not shown, manuscript in preparation). Interestingly, after normalizing nicotine intake (TNE-adjusted), EC users had a significantly higher level of PMA (for benzene) compared to SM, although the mean difference was small. Overall, TNE-adjusted VOCs among EC users compared to SM had larger standard deviations. This may be due to a wide range of nicotine intake and the various EC types (Supplementary Table 6). Nonetheless, our findings add to the growing evidence that EC users are typically exposed to fewer VOCs than SM, but EC use may not be benign. There may be some residual effects of former smoking on mAge estimates and VOCs among EC users in the current study, but no pattern of former smoking history was observed (Supplementary Figs. 1–3). Thus, our findings suggest potential effects associated with EC-related VOCs, particularly acrylonitrile and 1,3-butadiene.

Although we did not observe significant overall associations between Cr-adjusted VOCs and epigenetic aging after adjusting for smoking status, we found significant associations with TNE-adjusted VOCs. Typically, urinary biomarker studies report Cr-adjusted metabolites of VOCs to assess their exposure in clinical settings. However, in the current study, different findings on the relationship between VOCs and biological effects—between with/without nicotine intake—suggest that future research needs to consider carefully controlling for nicotine consumption. This is important because the varying levels of nicotine intake among SM and EC users may influence the biological implications of VOC exposure, as most of the VOC metabolites are significantly correlated with nicotine consumption (Supplementary Table 7). Thus, without controlling for nicotine intake, which is the current standard, it is likely that findings may underestimate or not identify potential biological implications.

Using TNE-adjusted metabolites of VOCs, we found overall older and accelerated Pheno-mAge in relation to higher levels of CNEMA (for acrylonitrile), HEMA (for ethylene oxide), and HMBMA1,3 (for 1,3-butadiene), after controlling for smoking group, along with significant interactions of the associations by the smoking group. According to the International Agency for Research on Cancer (IARC), these VOCs are determined as carcinogenic to humans (Group 1) based on “sufficient” evidence for cancer from studies in humans, including epidemiological and mechanistic studies (Stayner et al., 2024; Anon, 2001; Triangle Park, 2021). Regarding the biological impacts of these VOCs, acrylonitrile can induce oxidative stress and cause immortalization by inducing senescence-related genes(Anon, 2001). Ethylene oxide is a highly reactive VOC that can cause lung damage and is associated with increased pulmonary disease risks (Huang et al., 2023). Exposure to 1,3 butadiene can cause irritation and inflammation of respiratory tracts (Nellis et al., 2021). Pheno-mAge (i.e., phenotypic epigenetic age as a marker for lifespan and health span) (Levine et al., 2018), is related to increased inflammatory pathways and decreased damage recognition and repair pathways (Levine et al., 2018), further studies are needed to determine the potential contribution of these VOCs to Pheno-mAge and CpGs captured by Pheno-mAge in pulmonary diseases. This is especially important among SM as within-group analyses indicate a significant association in this group. Interestingly, given that most of the EC users in the current study are former smokers (Supplementary Table 6), different association patterns between SM and EC indicate the potential reversibility of methylation at CpGs used to estimate Pheno-mAge. Further studies are needed in the longitudinal research setting to capture the potential changes of Pheno-mAge over time based on the use of EC patterns and changes in EC device features to determine the unique contributions of EC to biological aging.

In addition to mAge estimates, we examined the relationships between metabolites of smoking and EC-related VOCs and senescence-related gene expression (Avelar et al., 2020; Chatsirisupachai et al., 2019). Independent of smoking status, TNE-adjusted metabolites analyses revealed that higher *IFNG* expression levels are associated with higher levels of MMA (for methylating agent), 2HPMA (for propylene oxide), and AAMA (acrylamide), while higher levels of *STAT1* expression were associated with higher levels of AAMA. Both *IFNG* and *STAT1* play a role in inflammation. Specifically, *IFNG*, interferon-gamma, plays a key role in the immune defense and activates immune cells, including macrophages (Casanova et al., 2024). In healthy individuals, expression of this gene generally increases with aging (Bandres et al., 2000; Kim et al., 2009). *STAT1* is a transcription factor that mediates immune responses and *STAT1* deficiency causes airway dysfunction and increase of mucus, resulting in predisposing highly susceptible individuals to pulmonary viruses and pathogenic infection (Hashimoto et al., 2005; Hongerholt et al., 1997). Interestingly, we observed significant interaction effects of the associations by smoking group with different patterns of the associations between SM and EC users, supporting further studies to understand the different biological implications of VOCs-related cell senescence genes by smoking group in pulmonary diseases.

Interestingly, from Cr-adjusted VOCs analyses after adjusting for smoking group, we observed an overall positive association of PMA (for benzene) associated with *POU5F1*, particularly among SM observed in the within-group analysis. *POU5F1* encodes a transcription factor protein (i.e., Oct-4) controlling stem cell pluripotency, and is known to enhance the invasiveness of cancer stem-like cells in lung cancer (Xin et al., 2013). We did not find this relationship after adjusting for nicotine intake, which indicates that this relationship may be nicotine dependent. Given the important role of benzene in disease pathogenesis and a higher level of TNE-adjust benzene in EC users, future studies are warranted to determine the potential contributions of benzene-related pulmonary diseases, possibly mediated by cellular senescence-related genes in EC users. One of the main strengths of our study was to utilize the target organ of smoking and EC exposure to address the potential implications of exposure to the wide range of VOCs on lung aging. We also conducted the analyses with and without considering nicotine intake, controlled for the smoking group to understand overall associations, and explored potentially different impacts of the exposure between SM and EC users using an interaction effect in our analytical models. Moreover, we studied lung biological aging not only at epigenetic levels but also at transcription levels (i.e., cellular senescence-related gene expression). However, there are also important limitations to be addressed. As there is no lung tissue-specific mAge available and all mAge estimates except Horvath-mAge were developed based on blood DNA methylation, the estimated lung mAge may not reflect accurate biological aging. Thus, our focus was to understand the associations of mAge estimates with VOCs, independent of chronological age with VOCs, rather than predicted mAge itself. While we focused on four well-studied epigenetic clocks, there may be other mAge estimates that could also be associated with VOCs. Our cross-sectional study design limits our ability to draw causality from these findings. As VOCs lack sensitivity in identifying exposure to smoking/EC-related constituents, there is also the potential for unknown or unrecorded covariates factors (e.g., ambient and indoor air pollution, occupation, social determinants of health-related factors, etc) to mediate the associations we do see. Also, given the small sample size, larger studies are required to validate our findings, with the expansion of comparison groups including former smokers with and without EC use and dual users of EC and cigarettes or in longitudinal study designs.

In summary, this is the first study showing the associations between smoking and EC-related VOCs and lung biological aging, supporting further research on the roles of VOC-related epigenetic aging and cell senescence genes in pulmonary diseases. Our findings suggest that smoking and EC-related VOC exposure-driven pulmonary aging may increase vulnerability to respiratory diseases and a greater risk of chronic lung diseases. With the reversible nature of DNA methylation, epigenetic-age biomarkers, especially Pheno-mAge, could provide insights into the long-term health effects of changing exposure to VOCs by quitting and switching to another product, if confirmed in more accessible tissues.

## Supplementary Material

MMC10

MMC9

MMC6

MMC7

MMC8

MMC5

MMC4

MMC1

MMC3

MMC2

Supplementary data to this article can be found online at https://doi.org/10.1016/j.scitotenv.2025.179792.

## Figures and Tables

**Table 1:** Characteristics of study participants.

Characteristics	Participants			
	Overall ( *n* = 56)	Smoker (n = 13)	EC user (n = 12)	Never Smoker (n = 31)
Age (Years), mean (SD)	26.4 (2.6)	26.7 (2.6)	27.0 (2.2)	26.0 (2.7)
Gender, n (%)				
Female	24 (42.1 %)	2 (15.4 %)	4 (30.8 %)	18 (58.1 %)
Male	33 (57.9%)	11 (84.6%)	9 (69.2%)	13 (41.9%)
Race, n (%)				
White	45 (78.9%)	12 (92.3%)	10 (76.9%)	23 (74.2%)
Non-White	12 (21.1%)	1 (7.7%)	3 (23.1%)	8 (25.8%)
Smoking				
Years of smoking, median (range)	–	9 (0.6–13)	7 (2–11)*	–
Cigarette per day, median (range)	–	20 (10–20)	15 (2–20)*	–
EC use				
Years of EC use, median (range)	–	–	3 (1–4)	–
Puff per day, median (range)	–	–	100 (20–600)	
EC-liquid (ml) per day, median (range)	–	–	7.5 (2–20)	–
TNE (pg/mg Cr), median (range)	0.02 (0.0008–68.6)	19.5 (5.5–68.6)	15.9 (0.1–62.4)	0.003 (0.0008–0.7)
Propylene Glycol (mg/ml), median (range)	3.68 (0.15–221.4)	6.6 (1.2–50.3)	28.5 (1.5–75.1)	1.98 (0.2–221.4)

EC, electronic cigarette; n, number;

* Prior smoking EC users; TNE; Total nicotine equivalents (Cotinine +3-hydroxycotinine).

**Table 2:** Summary statistics of total nicotine intake-adjusted urinary biomarkers in smokers and EC users.

Urinary metabolite	Parent compound	Total nicotine equivalents-adjusted volatile organic compounds				
		Mean (SD)			Fold difference	
		Overall ( *n* = 25)	EC (n = 12)	SM (n = 13)	SM/EC	*P*-value
AAMA	Acrylamide	21.92 ± 55.54	33.53 ± 79.70	11.15 ± 6.83	0.3	0.89
CNEMA	Acrylonitrile	6.34 ± 5.57	**4.50 ± 6.98**	**8.05 ± 3.24**	**1.8**	**1.00E-03**
HEMA	Ethylene oxide	0.41 ± 0.97	0.64 ± 1.39	0.19 ± 0.09	0.3	0.23
HPMMA	Crotonaldehyde	58.90 ± 190.22	103.50 ± 272.13	17.48 ± 13.19	0.2	0.67
MHBMA1,2	1,3-butadiene	0.12 ± 0.10	**0.08 ± 0.12**	**0.14 ± 0.08**	**1.7**	**8.00E-03**
MHBMA3	1,3-butadiene	0.02 ± 0.05	0.04 ± 0.08	0.01 ± 0.01	0.2	0.40
MMA	Methylating agent	8.72 ± 35.22	17.40 ± 50.29	0.66 ± 0.67	0.0	0.42
PMA	Benzene	0.09 ± 0.17	**0.11 ± 0.24**	**0.08 ± 0.07**	**−1.3**	**2.50E-02**
2-HPMA	Propylene oxide	22.11 ± 62.43	38.65 ± 88.40	6.75 ± 7.32	0.2	0.71
3-HPMA	Acrolein	164.11 ± 433.83	272.63 ± 616.62	63.35 ± 52.36	0.2	0.32

Means and fold differences were summarized using raw data for easier interpretation while statistical analyses were conducted using log10-transformed data for normality.

Data in bold are significant at *P <* 0.05 (two-sample t-test).

EC, electronic cigarette; NS, never smoker; SM, smoker.

*N*-acetyl-S-(2-carbamoylethyl)-L-cysteine (AAMA), cyanoethylmercapturic acid (CNEMA), 2-hydroxyethylmercapturic acid (HEMA), 3-hydroxy-1-methylpropylmercapturic acid (HPMMA), 2-hydroxy-3-buten-1-yl-mercapturic acid (MHBMA1,2), 4-hydroxy-2-buten-1-yl-mercapturic acid (MHBMA3), methyl mercapturic acid (MMA), phenyl mercapturic acid (PMA), 2-hydroxypropyl mercapturic acid (2-HPMA), 3-hydroxypropyl mercapturic acid (3-HPMA).

Unit, ng/nmol.

## HIGHLIGHTS

- Smokers had higher TNE-adjusted metabolites of acrylonitrile and 1,3-butadiene but lower benzene metabolite than EC users.
- TNE-adjusted metabolites of 1,3 butadiene, ethylene oxide, and acrylonitrile were positively associated with Pheno-mAge.
- Age-related genes, *IFNG* and *STAT1*, were associated with TNE-adjusted metabolites of propylene oxide and acrylamide.
- The associations of TNE-adjusted metabolites or age-related genes and Pheno-mAge found to differ between SM and EC users.^2^
