# Supplementary figures and images for: Associations of volatile organic compounds with accelerated epigenetic aging in the lungs of smokers and electronic cigarette users
Source: Sci Total Environ. Author manuscript; Available in PMC 2026 Jun 30. (PMC13318086; doi:10.1016/j.scitotenv.2025.179792)

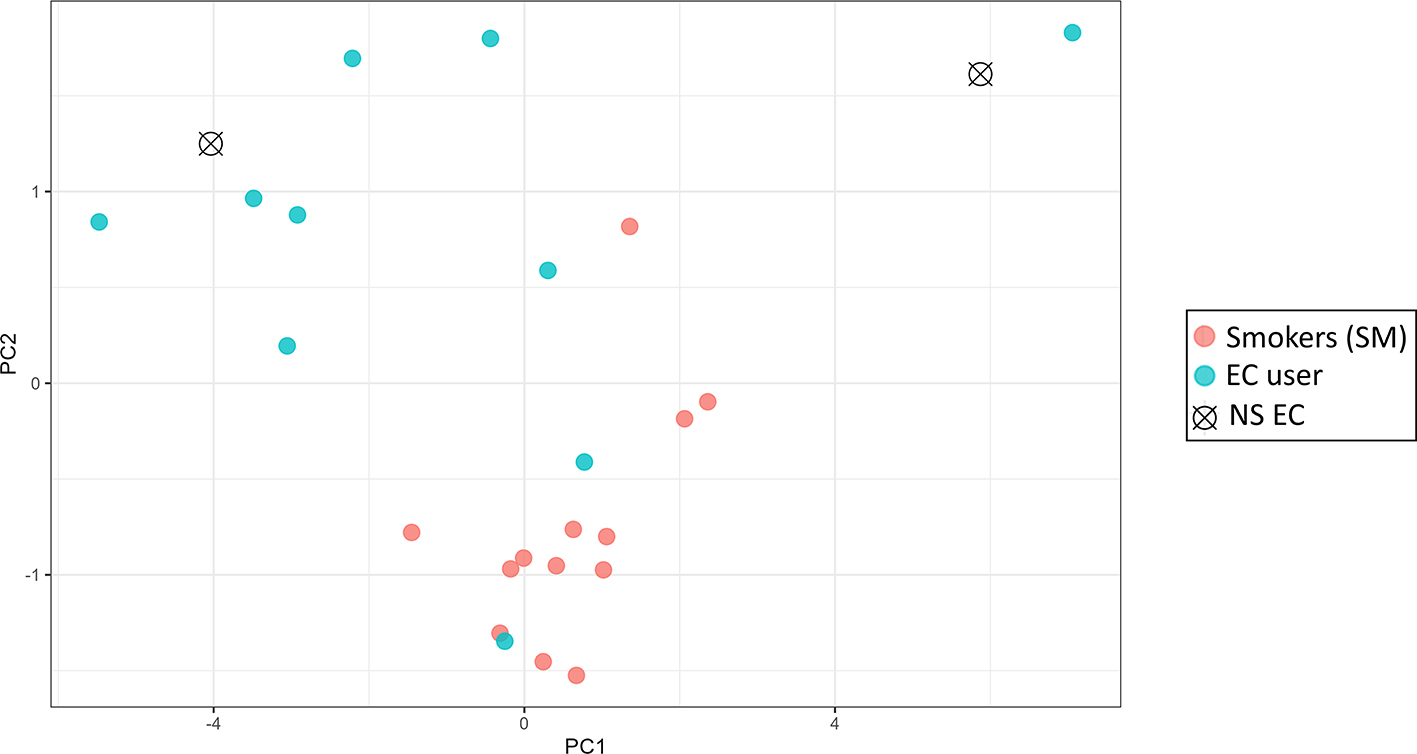

Supplement: MMC1 [file NIHMS2176749-supplement-MMC1.jpg]

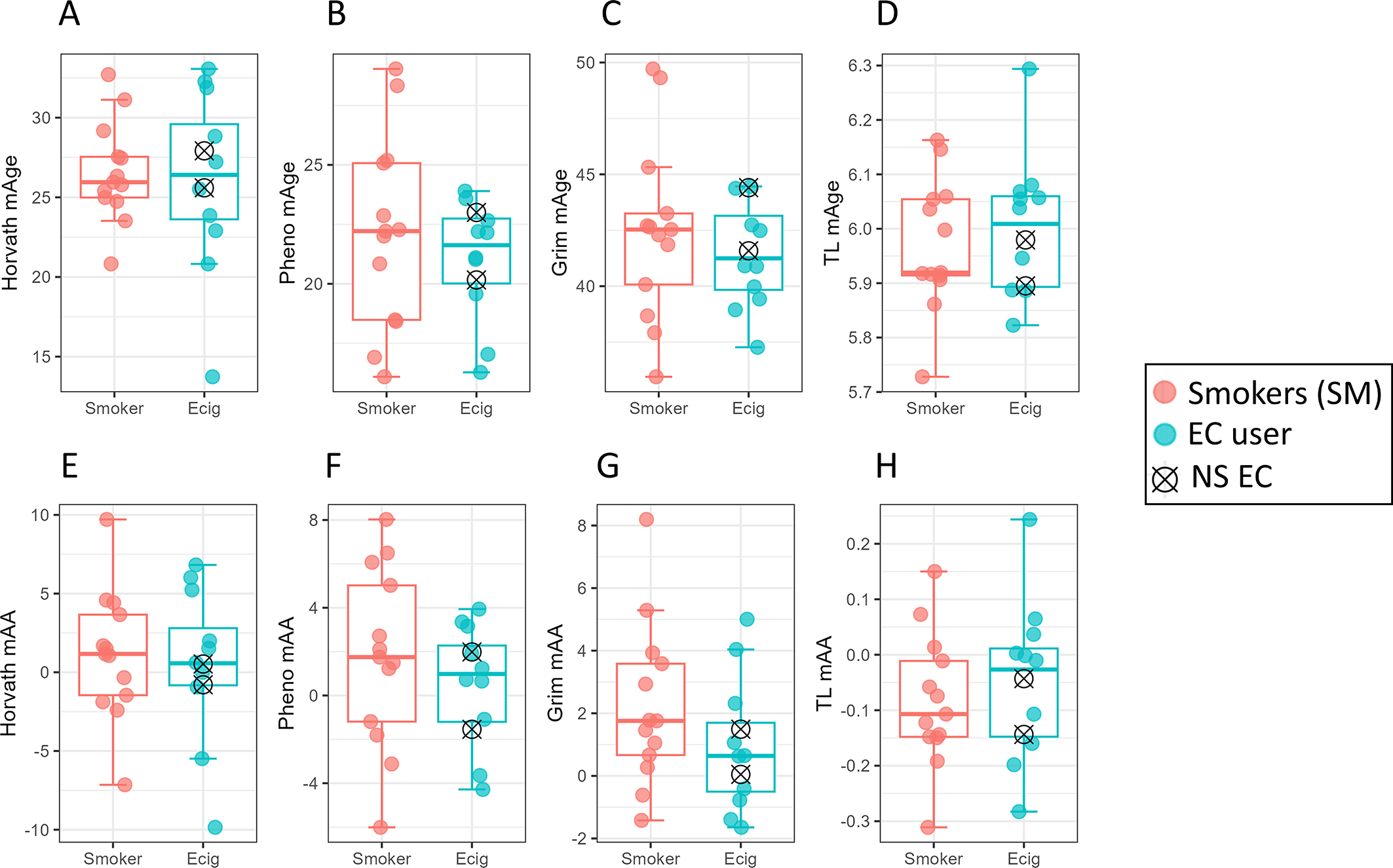

Supplement: MMC3 [file NIHMS2176749-supplement-MMC3.jpg]

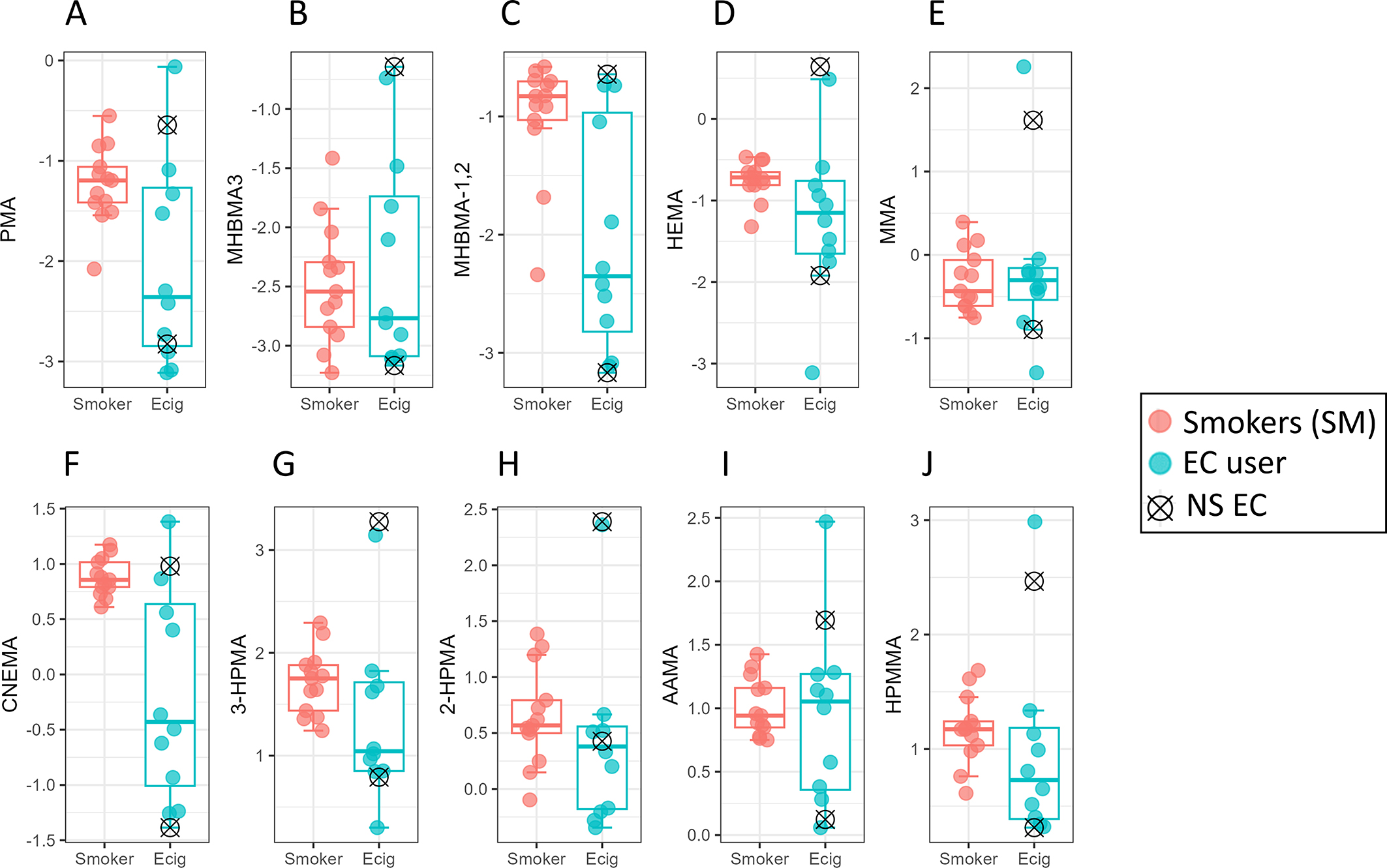

Supplement: MMC2 [file NIHMS2176749-supplement-MMC2.jpg]
